# Supplementary figures and images for: Personalized analysis of breast cancer using sample-specific networks
Source: PeerJ. 2020 May 15;8:e9161. doi: 10.7717/peerj.9161 (PMC7233277; doi:10.7717/peerj.9161)

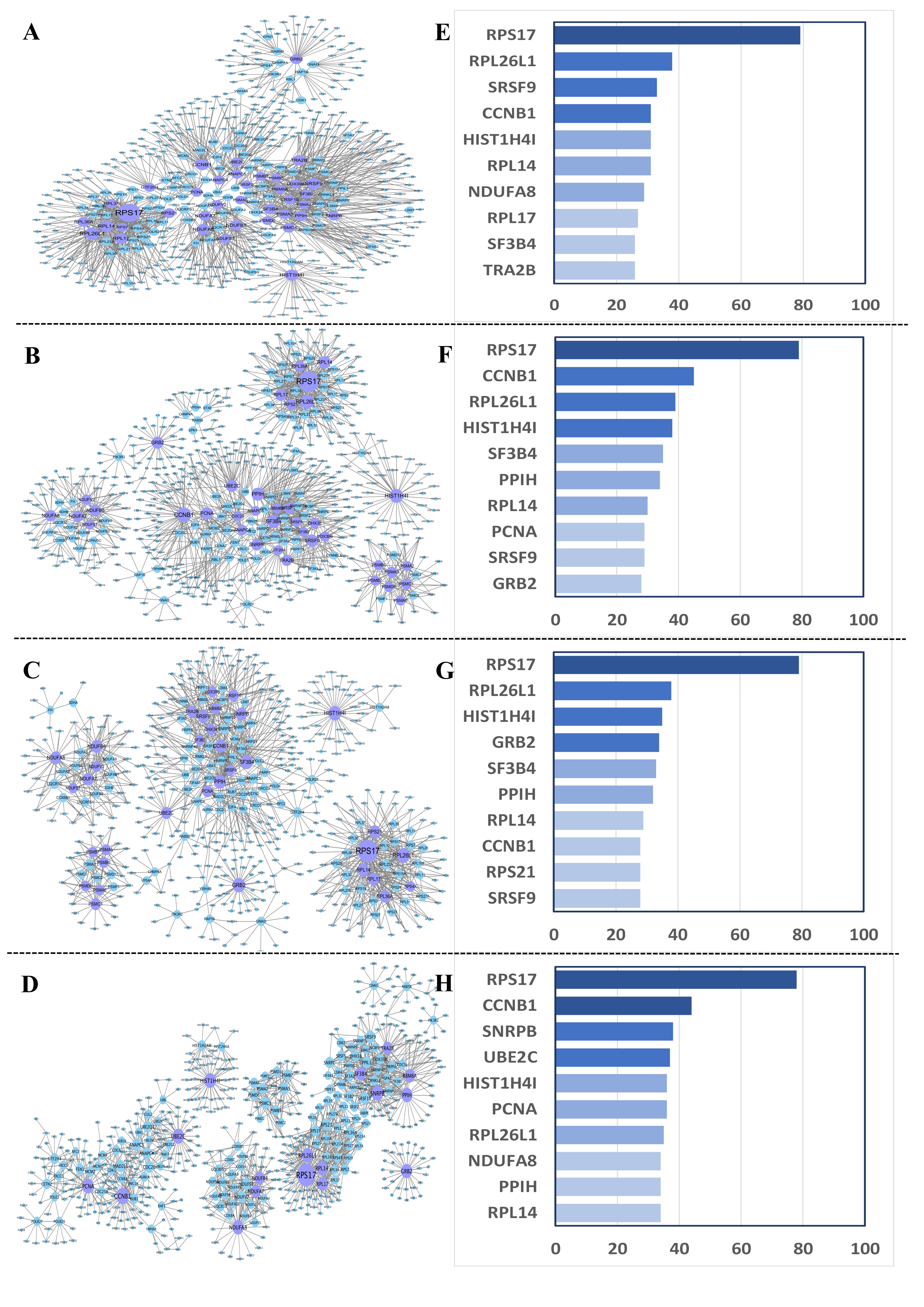

Supplement: Supplemental Information 1 — <!--[if !supportLists]-->(A-<!--[endif]-->D) Gene-gene interaction networks associated with Stage I, II, III, and IV respectively. (E-H) The bar charts of top 10 genes with the highest degrees in gene–gene interaction networks related to Stage I, II, III, and IV. The Y axis is gene, and the X axis is the gene degree. [file peerj-08-9161-s001.png]

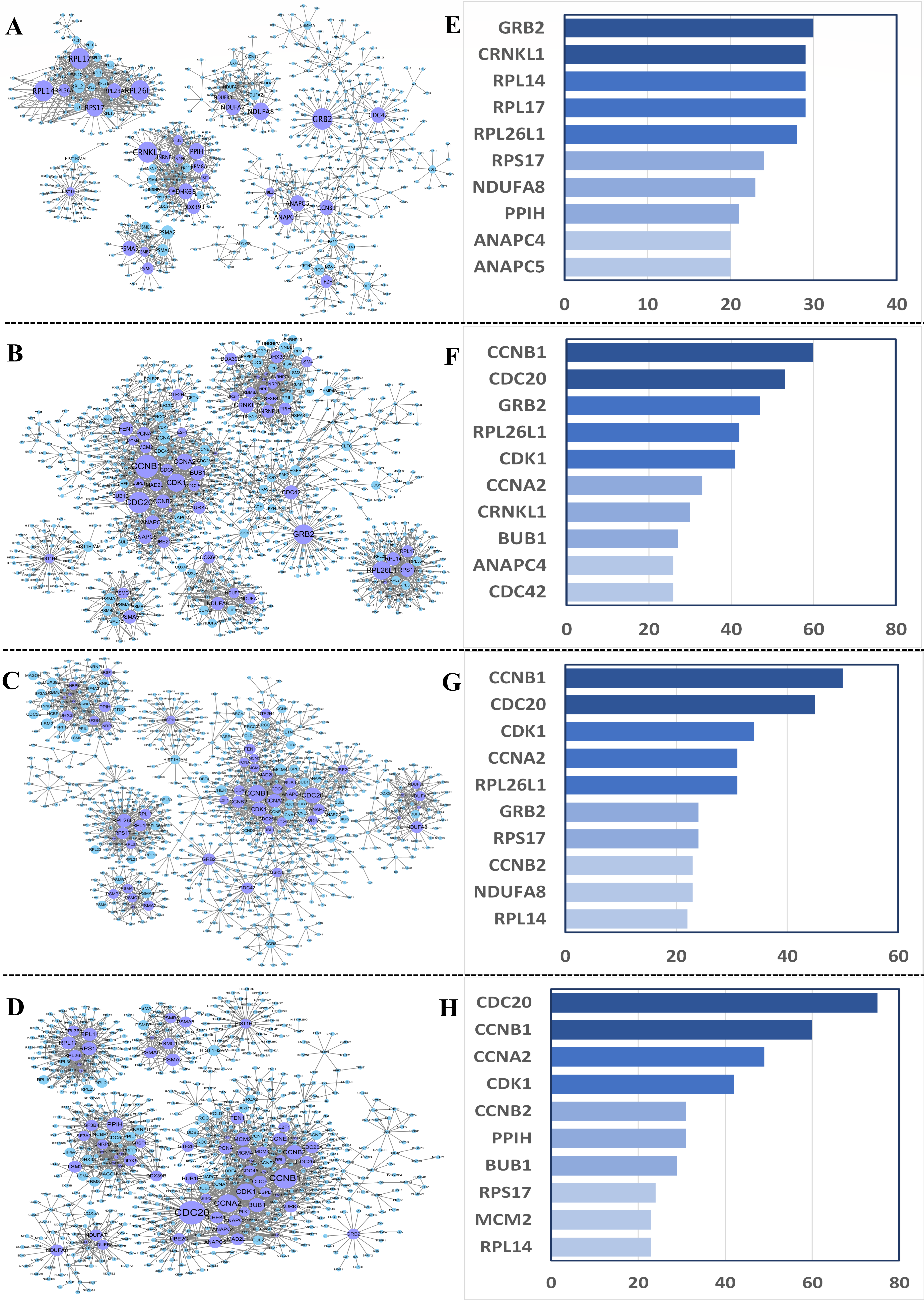

Supplement: Supplemental Information 2 — <!--[if !supportLists]-->(A–D) Gene-gene interaction networks associated with LumA, LumB, Her2, and Basal-like subtypes respectively. (E–H) The bar charts of top 10 genes with the highest degrees in gene–gene interaction networks related to LumA, LumB, Her2, and Basal-like subtypes. The Y axis is gene, and the X axis is the gene degree. [file peerj-08-9161-s002.png]

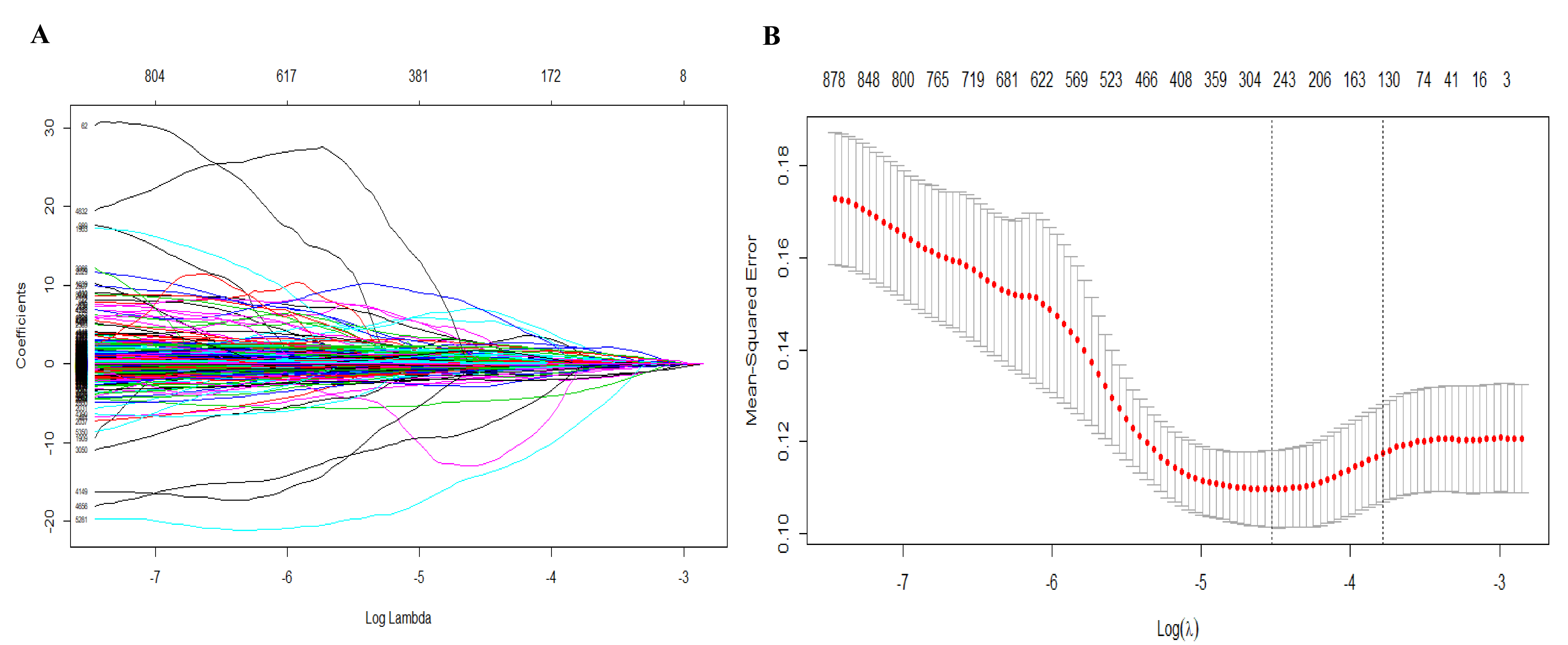

Supplement: Supplemental Information 3 — (A) Four-fold cross-validation for tuning parameter (λ) selection in the LASSO model. (B) LASSO coefficient profiles of 272 gene interactions. [file peerj-08-9161-s003.png]
